# Predictive Fat Mass Equations for Children With Inflammatory Bowel Disease

**DOI:** 10.1097/MPG.0000000000003188

**Published:** 2021-06-04

**Authors:** Francesca Penagini, Alessandro Leone, Barbara Borsani, Alessandra Bosetti, Dario Dilillo, Giulia Rendo, Valeria Calcaterra, Simona Bertoli, Stefano Mora, Alberto Battezzati, Giorgio Bedogni, Gian Vincenzo Zuccotti

**Affiliations:** ∗ Department of Pediatrics, “V. Buzzi” Hospital; † International Center for the Assessment of Nutritional Status (ICANS), Department of Food, Environmental and Nutritional Sciences; ‡ Department of Pediatrics, “V. Buzzi” Children's Hospital, University of Milan, Milan; § Pediatric and Adolescent Unit, Department of Internal Medicine, University of Pavia, Pavia; ¶ Pediatric Bone Densitometry Service and Laboratory of Pediatric Endocrinology, IRCCS San Raffaele Institute, Milan; || Clinical Epidemiology Unit, Liver Research Center, Basovizza, Trieste, Italy.

**Keywords:** body composition, nutrition, paediatric inflammatory bowel disease

## Abstract

**Objective:**

Evaluate accuracy of skinfold thicknesses and body mass index (BMI) for the prediction of fat mass percentage (FM%) in paediatric inflammatory bowel disease (IBD) and to develop population-specific formulae based on anthropometry for estimation of FM%.

**Methods:**

IBD children (n = 30) and healthy controls (HCs, n = 144) underwent anthropometric evaluation and dual-energy X-ray absorptiometry (DEXA) scan, as the clinical reference for measurement of body composition. Body FM% estimated with skinfolds thickness was compared with FM% measured with DEXA. By means of 4 prediction models, population specific formulae for estimation of FM% were developed.

**Results:**

No significant difference in terms of FM% measured by DEXA was found between IBD population and HCs (FM% 29.6% vs 32.2%, *P* = 0.108). Triceps skinfold thickness (TSF, Model 2) was better than BMI (Model 1) at predicting FM% (82% vs 68% of variance). The sum of 2 skinfolds (biceps + triceps; SF2, Model 3) showed an improvement in the prediction of FM% as compared with TSF, Model 2 (86% vs 82% of variance). The sum of 4 skinfolds (biceps + triceps + suprailiac + subscapular; Model 4) showed further improvement in the prediction of FM% as compared with SF2 (88% vs 86% of variance).

**Conclusions:**

The sum of 4 skinfolds is the most accurate in predicting FM% in paediatric IBD. The sum of 2 skinfolds is less accurate but more feasible and less prone to error. The newly developed population-specific formulae could be a valid tool for estimation of body composition in IBD population and an alternative to DEXA measurement.

## Abstract 2

Supplemental Digital Content is available in the text

**What Is Known/What Is New**

**What Is Known**

- Monitoring of nutritional status and body composition is important to prevent malnutrition and improve disease outcome in patients with inflammatory bowel disease.
- Dual-energy X-ray absorptiometry, the clinical gold standard for body composition analysis, is not always available in clinical practice.

**What Is New**

- In this study, we developed population-specific formulae based on anthropometry for estimation of fat mass percentage in paediatric inflammatory bowel disease as an alternative to dual-energy X-ray absorptiometry.
- The sum of 4 skinfolds is the most accurate in predicting fat mass percentage, the sum of 2 skinfolds is less accurate but more feasible in clinical practice because less prone to inter-operator error.

Inflammatory bowel disease (IBD) is a group of disorders characterized by chronic and relapsing inflammation of the gastrointestinal tract. The etiopathogenesis is multifactorial involving genetic predisposition and environmental factors (1,2). Nutrition is not only a key element in the pathogenesis of disease but also as an important factor influencing disease course; nutritional approach with exclusive enteral nutrition (EEN) and more recently specific diets, such as Crohn Disease Exclusion Diet (CDED) have been demonstrated to be effective for induction of remission in paediatric CD (3,4).

Weight loss, growth restriction, malnutrition, and bone mass deficit have been well described in paediatric IBD (5). Data on body composition in children and adolescents with IBD is scarce and discordant (6). Dual-energy X-ray absorptiometry (DEXA), the clinical gold standard for assessment of body composition, is not always available in clinical practice. Simple, reliable, rapid, and cost effective methods are needed for estimation of body composition in clinical practice. Prediction formulas for the estimation of FM% are available. These formulas have been created for the general population but are not disease-specific. Callias et al (7) evaluated the level of agreement between some plicometric equations (Deurenberg, Slaughter, Weststrate, Durnin and Rahaman, Johnston, Brook) and DEXA in a population of children and adolescents with IBD, concluding that although Durnin and Rahaman was found to have the best agreement with DEXA, none of the plicometric equations used was accurate enough to predict the amount of FM from plicometric measurements in paediatric IBD.

The objectives of the present study are:

1. Compare body composition of patients with IBD with healthy controls (HCs).
2. Evaluate accuracy of skinfold thicknesses and body mass index (BMI) for the prediction of FM% in children and adolescents with IBD by comparing results with FM% measured with DEXA.
3. Develop population-specific formulae based on anthropometry for estimation of FM% in paediatric IBD.

## METHODS

Thirty patients affected by IBD were prospectively recruited between September 2019 and May 2020 from the Gastroenterology Unit of “Vittore Buzzi” Children's Hospital, Milan, Italy. IBD patients were recruited at any time of their disease course. Inclusion criteria were: ages 6 to 18 years; diagnosis of Crohn disease (CD), ulcerative colitis (UC), or unclassified IBD (IBDU). Exclusion criteria were: age less than 6 or greater than 18 years old; diagnosis under definition. All subjects enrolled in the study underwent a complete nutritional assessment through clinical and instrumental evaluation, as described below. Informed consent was obtained from parents or legal guardian before participation in the study. At the moment of enrollment, data on disease location at diagnosis, disease activity, and medical treatment were collected. To define disease activity, the paediatric ulcerative colitis activity index (PUCAI score) (8) and the short paediatric Crohn disease activity index (sPCDAI) (9) was used, respectively for UC and CD.

HCs (n = 144) were children and adolescents ages 6 to 18 years attending the International Center for the Assessment of Nutritional Status (ICANS, University of Milan) for screening of nutritional status. All control children and adolescents underwent anthropometric measurement and DEXA. To be eligible for the study, they had to be free of known acute (eg, influenza) and chronic disease (eg, diabetes). Informed consent was obtained from each patient's and HC's legal guardian before enrollment.

### Anthropometric Evaluation

In IBD patients, the following parameters were measured: weight, height, pubertal stage, BMI, body circumferences (waist, abdomen, hips), and skinfold thicknesses (biceps, triceps, suprailiac, and subscapular skinfolds). Body weight was measured to the nearest 100 g with a beam scale, and body height to the nearest 0.1 cm using a vertical stadiometer. For pubertal stages, we considered Prepubertal stage = Tanner Stage 1; Middle puberty = Tanner Stage 2 and 3; Late puberty = Tanner Stage 4 and 5 (10,11). BMI was calculated as weight (kg)/height (m^2^). The standard deviation scores (SDS) of weight, height, and BMI were calculated using WHO reference data (12). Nutritional status was defined using the International Obesity Task Force (IOTF) reference (13). Skinfold thicknesses were measured using a professional mechanical skinfold caliper (GIMA). Each skinfold was measured 3 times, and the mean value was considered and recorded to the nearest 0.1 mm. Measurements were collected by trained dietitians using a standardized technique (14).

### Estimation of Body Fat Mass Percentage

Predictive equations based on skinfold thicknesses were used for estimation of body FM %.

The following formulas were used to calculate body density:

1. Brook (15): 1 to 9 years old
  a. Girls: body density = 1.2063 − 0.0999 × (log Σ of the 4 skinfolds)
  b. Boys: body density = 1.1690 – 0.0788 × (log Σ of the 4 skinfolds)
2. Johnston et al (16): 8 to 14 years old
  a. Girls: body density = 1.144 – 0.06 × (log Σ of the 4 skinfolds)
  b. Boys: body density = 1.166 – 0.07 × (log Σ of the 4 skinfolds)
3. Durnin and Rahaman (17): 13 to 15 years old
  a. Girls: body density = 1.1369 – 0.0598 × (log Σ of the 4 skinfolds)
  b. Boys: body density = 1.1533 – 0.0643 × (log Σ of the 4 skinfolds)
4. Durnin and Womersley (18): 16 to 19 years old
  a. Girls: body density = 1.1549 – 0.0678 × (log Σ of the 4 skinfolds)
  b. Boys: body density = 1.162 – 0.063 × (log Σ of the 4 skinfolds)

The value of body density (D) obtained, which is inversely related to fat content of the body, was used to estimate the FM (Fat Mass) through Siri predictive equation (19):

5. Siri Equation: FM (%) = 495/D – 450.

### Body Composition Evaluation With Dual X-ray Absorptiometry Technique

Within 4 weeks from the nutritional assessment, all subjects with IBD underwent a body composition study with DEXA technique at the Pediatric Bone Densitometry Unit, San Raffaele Scientific Institute, using the Lunar Prodigy Advance DEXA System—GE Medical Systems LUNAR (software version 16). In IBD patients, FM% estimated with the above mentioned available predictive formulae (Brook, Johnston, Durnin and Rahaman, Durnin and Womersley) was compared with FM% measured with DEXA scan.

### Statistical Analysis

Most continuous variables were not Gaussian-distributed, and all are reported as 50th (median), 25^th^, and 75th percentiles. Discrete variables are reported as the number and proportion of subjects with the characteristic of interest. Between-group comparisons were performed with the Wilcoxon Mann-Whitney test for continuous variables and with the Pearson's chi-squared test for discrete variables. We evaluated the contribution of BMI, triceps skinfold (TSF), the sum of 2 skinfolds (triceps + biceps), and the sum of 4 skinfolds (triceps + biceps + subscapular + suprailiac) to percent fat mass (FM%), that is, fat mass (FM, kg)/body mass (BM, kg) using 2 prespecified linear regression models. The response variable of both models was FM% (%). The predictors of Model 1 were logeBMI (mm), logeTSF (mm), loge2SF (mm) or loge4SF (mm), IBD status (discrete, 0 = CTR; 1 = IBD) and their interaction (continuous X discrete). All the predictors were loge transformed to ensure homoskedasticity (20). If the interaction of Model 1 was not significant, that is, the regression lines of CTR and IBD were parallel, we evaluated Model 2, that is, Model 1 without the interaction term. Model 2 tests the hypothesis that the parallel regression lines detected by Model 1 are superimposed. To control for potential confounding factors, both models were adjusted for sex (discrete; 0 = female, 1 = male) and age (continuous). The linearity of the predictor X IBD interaction was checked using plots and multivariable fractional polynomials (21). Standard diagnostic plots were used to evaluate model fit. The adjusted coefficient of determination (*R*^2^_adj_) and the root mean squared error of the estimate (RMSE) were used as measures of model fit (22). The 95% confidence intervals (95% CI) of the regression coefficients, *R*^2^_adj_ and RMSE were calculated using bootstrap on 1000 random samples of 174 subjects, that is, the whole sample (23). The bootstrap offers an efficient way of correcting for overoptimism and is presently considered the best method for performing internal cross-validation (24).

With regards to comparison between FM% estimated with predictive formulae and FM% measured with DEXA, Bland and Altman method was used to calculate the limits of agreement (LOA) between predicted and measured FM%. Bias was calculated as (predicted FM% − measured FM%) and percentage bias as [(predicted FM% − measured FM%)/measured FM%] × 100. Pitman's test was used to evaluate proportional bias. Statistical analysis was performed using Stata 15.1 (Stata Corporation, College Station, TX).

## RESULTS

Demographic, anthropometric, and body composition data of our study population are shown in Table 1. Of all the IBD patients, 16 had CD (53.3%), 12 UC (40%), 2 IBD-U (6.6%). In IBD patients, median age at recruitment was 14 years (interquartile range 11--16). F : M ratio was 10 : 20. Pubertal stage was prepubertal in 16.6% (5/30), middle puberty in 33.3% (10/30), late puberty in 50% (15/30). Mean duration of disease at the moment of enrollment was 21 months (± 9 months). Data on disease location, activity, and treatment of IBD patients are shown in Table, Supplementary Digital Content, http://links.lww.com/MPG/C366. According to the IOTF growth charts for BMI, 2 (6.7%) of IBD children had grade 1 thinness, 21 (70%) were of normal weight, 4 (13.3%) were overweight, and 3 (10%) were obese.

**TABLE 1:** Demographic, anthropometric, and body composition data of inflammatory bowel disease patients and healthy controls

	Total	HC	IBD	
	N = 174	N = 144	N = 30	
	median (IQ)	median (IQ)	median (IQ)	*P* value
Age, year	15 (13--16)	15 (14--17)	14 (11--16)	0.23
Weight, kg	59.8 (50.6--66.2)	60.5 (52.6--66.6)	51 (40.8--63.1)	0.004
Height, m	1.63 (1.56--1.69)	1.63 (1.58--1.69)	1.59 (1.45--1.69)	0.077
Height (SDS WHO)	0.245 (−0.273 to 0.819)	0.249 (−0.243 to 0.867)	0.195 (−0.501 to 0.697)	0.43
BMI, kg/m^2^	22.1 (19.9--24)	22.4 (20.3--24.1)	20.1 (17.5--22.4)	0.002
BMI (SDS WHO)	0.588 (−0.118 to 1.053)	0.61 (−0.083 to 1.052)	0.483 (−0.167 to 1.088)	0.503
Fat mass DEXA, kg	17.1 (12.6--22.7)	18.3 (13.5--23.4)	12.4 (8.6--17)	0.002
Fat mass DEXA, % of body mass	31.6 (25.4--36.5)	32.2 (25.8-- 36.7)	29.6 (21.7--34.9)	0.108
Biceps skinfold, mm	9.8 (6.6--13)	9.9 (6.7--13.1)	9.2 (5.4--12.8)	0.541
Triceps skinfold, mm	18.1 (12.8--24)	19.5 (13.8--24.1)	14 (11.2--18)	0.026
Subscapular skinfold, mm	13.6 (9.8--21.6)	14.8 (10.6--22.4)	8.7 (6.8--13)	<0.001
Suprailiac skinfold, mm	22.9 (13.6--33)	26.6 (16.6--35)	10.6 (7.8--15)	<0.001
Sum of 2 skinfolds, mm	33.1 (22.5--44.2)	35.5 (24.9--45.1)	23.1 (17.6-- 33)	<0.001
Sum of 4 skinfolds, mm	64.9 (43.4--90.6)	71.6 (48.9--92.6)	42.8 (31.4--55.6)	<0.001

Continuous variables. BMI = body mass index; DEXA = dual-energy X-Ray absorptiometry; FM = fat mass; HC = healthy controls; IBD = inflammatory bowel disease; IQ = interquartile range; SDS = standard deviation scores.

At the moment of recruitment, the control group (HCs = 144) had a median age of 15 years (interquartile range 14--17 years), a F : M ratio of 25 : 119. Pubertal stage was pre-pubertal in 18.7% (27/144), middle puberty in 9.7% (14/144), and late puberty in 71.5% (103/144). According to the IOTF growth charts for BMI, 5 (3.5%) had grade 1 thinness, 107 (74.3%) were normal weight, 17 (11.8%) were overweight, and 15 (10.4%) were obese.

**FIGURE 1:**
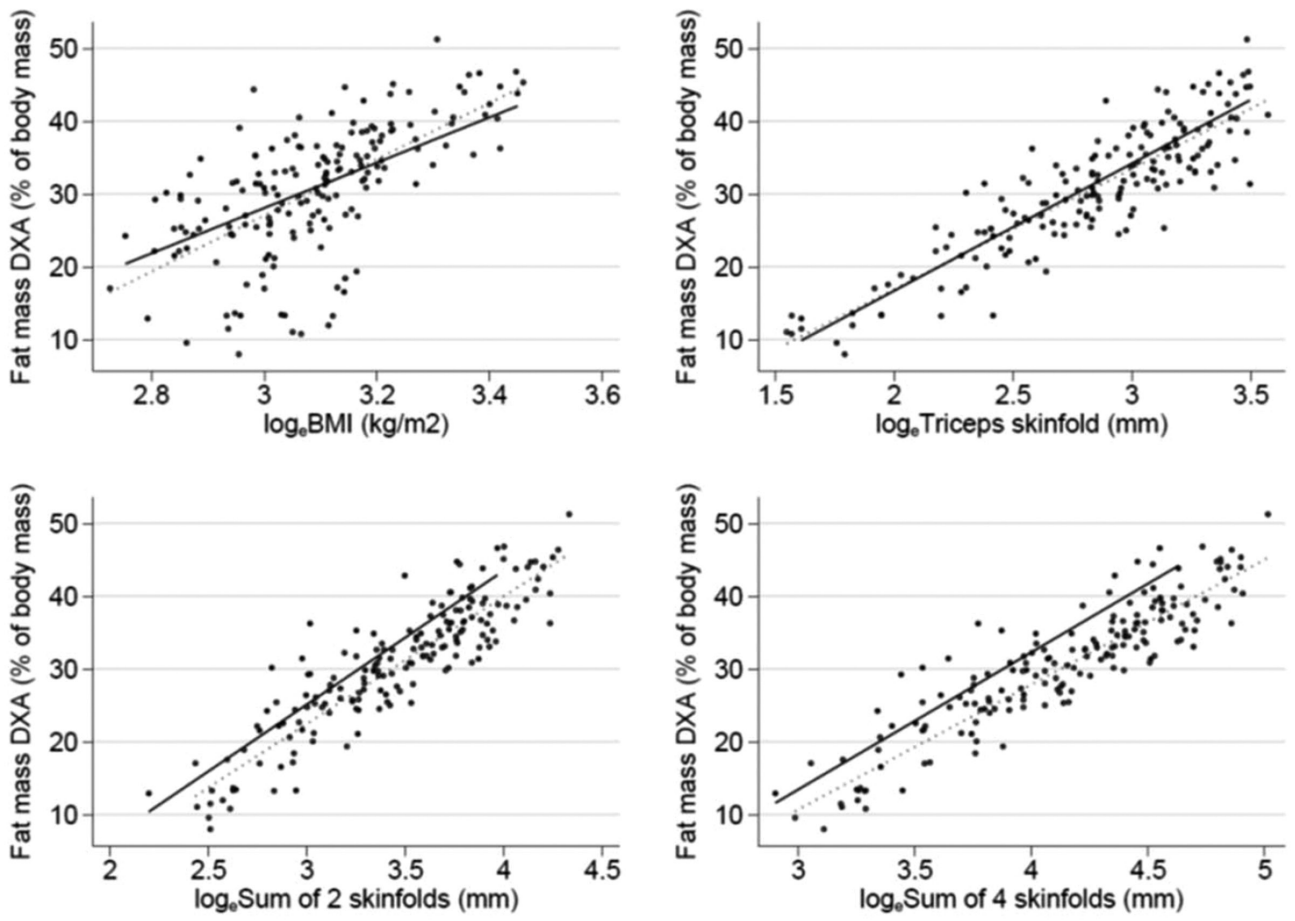
Scatterplots of fat mass percentage versus logeBMI, LogeTSF, LogeSF2, and LogeSF4.

The regression models used to predict FM% from anthropometry are given in Table 2. Triceps skinfold thickness (TSF, Model 2) was much better than BMI (Model 1) at predicting FM%, explaining 82% versus 68% of its variance and being associated with a RMSE of 3.8% versus 4.9%. The sum of 2 skinfolds (biceps + triceps, SF2- Model 3) offered a marginal improvement in the prediction of FM% as compared with TSF (Model 2), explaining 86% versus 82% of the variance of FM% and being associated with a RMSE of 3.2% versus 3.8%. The sum of 4 skinfolds (triceps + biceps + subscapular + suprailiac, SF4- Model 4) offered an even more modest improvement in the prediction of FM% as compared with SF2 (Model 3), explaining 88% versus 86% of the variance of FM% and being associated with a RMSE of 3.0% versus 3.2%. Figure 1 gives the scatterplots of FM% versus logeBMI, logeTSF, logeSF2, and logeSF4. All the relationships were linear and the predictor × IBD interaction was not significant in any model (data not shown).

**TABLE 2:** The 4 regression models used to predict fat mass percentage from anthropometry

	Model 1	Model 2	Model 3	Model 4
Sex (male)	−8.59^∗∗∗^ [−10.66 to −6.53]	−1.19 [−2.86 to 0.48]	−2.50^∗∗∗^ [−3.84 to −1.16]	−2.55^∗∗∗^ [−3.76 to −1.35]
Age, year	−1.50^∗∗∗^ [−1.82 to −1.18]	−0.77^∗∗∗^ [−1.01 to −0.53]	−0.77^∗∗∗^ [−0.98 to −0.57]	−0.66^∗∗∗^ [−0.85 to −0.47]
Log_e_BMI, kg/m^2^	36.73^∗∗∗^ [31.95--41.52]			
IBD	2.02 [−0.55 to 4.59]	−0.68 [−2.62 to 1.25]	2.13^∗^ [0.30--3.97]	3.58^∗∗∗^ [1.91--5.24]
Log_e_TSF		15.59^∗∗∗^ [14.20--16.97]		
Log_e_SF2			16.14^∗∗∗^ [15.02--17.26]	
Log_e_SF4				15.95^∗∗∗^ [14.91--16.99]
Constant	−58.35^∗∗∗^ [−72.83 to −43.87]	−1.39 [−7.47 to 4.68]	−12.97^∗∗∗^ [−18.47 to −7.47]	−25.06^∗∗∗^ [−30.87 to −19.25]
RMSE	4.94^∗∗∗^ [4.39--5.48]	3.75^∗∗∗^ [3.38--4.13]	3.23^∗∗∗^ [2.91--3.55]	3.03^∗∗∗^ [2.72--3.33]
*R* ^2^	0.68^∗∗∗^ [0.60--0.76]	0.82^∗∗∗^ [0.77--0.87]	0.86^∗∗∗^ [0.83--0.90]	0.88^∗∗∗^ [0.85--0.92]
N	174	174	174	174

95% confidence intervals in brackets. BMI = body mass index; IBD = inflammatory bowel disease; *R*^2^_adj_ = adjusted coefficient of determination; RMSE = root mean square error; SF = skinfold; TSF = triceps skinfold thickness.

∗ *P* < 0.05. ^∗∗^*P* < 0.01.

∗∗∗ *P* < 0.001.

### Population Specific Formulae For Estimation of FM% Based on Anthropometry

On the basis of the 4 prediction models, the proposed population-specific formulae for estimation of FM% based on anthropometry in paediatric patients with IBD are the following:

1. Formula based on BMI (derived from Model 1)
  FM% = −8.6 × sex (female = 0; male = 1) − 1.5 × age (years) + 2 × IBD (yes = 1, no = 0) + 36.7 × log_e_BMI − 58.4
2. Formula based on TSF (derived from Model 2)
  FM% = −1.2 × sex (female = 0; male = 1) − 0.8 × age (years) − 0.7 × IBD (yes = 1, no = 0) + 15.6 × log_e_TSF − 1.4
3. Formula based on 2 skinfolds (biceps + triceps; derived from Model 3)
  FM% = −2.5 × sex (female = 0; male = 1) − 0.8 × age (years) + 2.1 × IBD (yes = 1, no = 0) + 16.1 × log_e_SF2 − 13.0
4. Formula based on 4 skinfolds (biceps, triceps, subscapular + suprailiac; derived from Model 4)
  FM% = −2.6 × sex (female = 0; male = 1) − 0.7 × age (years) + 3.6 × IBD (yes = 1, no = 0) + 16.0 × log_e_SF4 − 25.1

**FIGURE 2:**
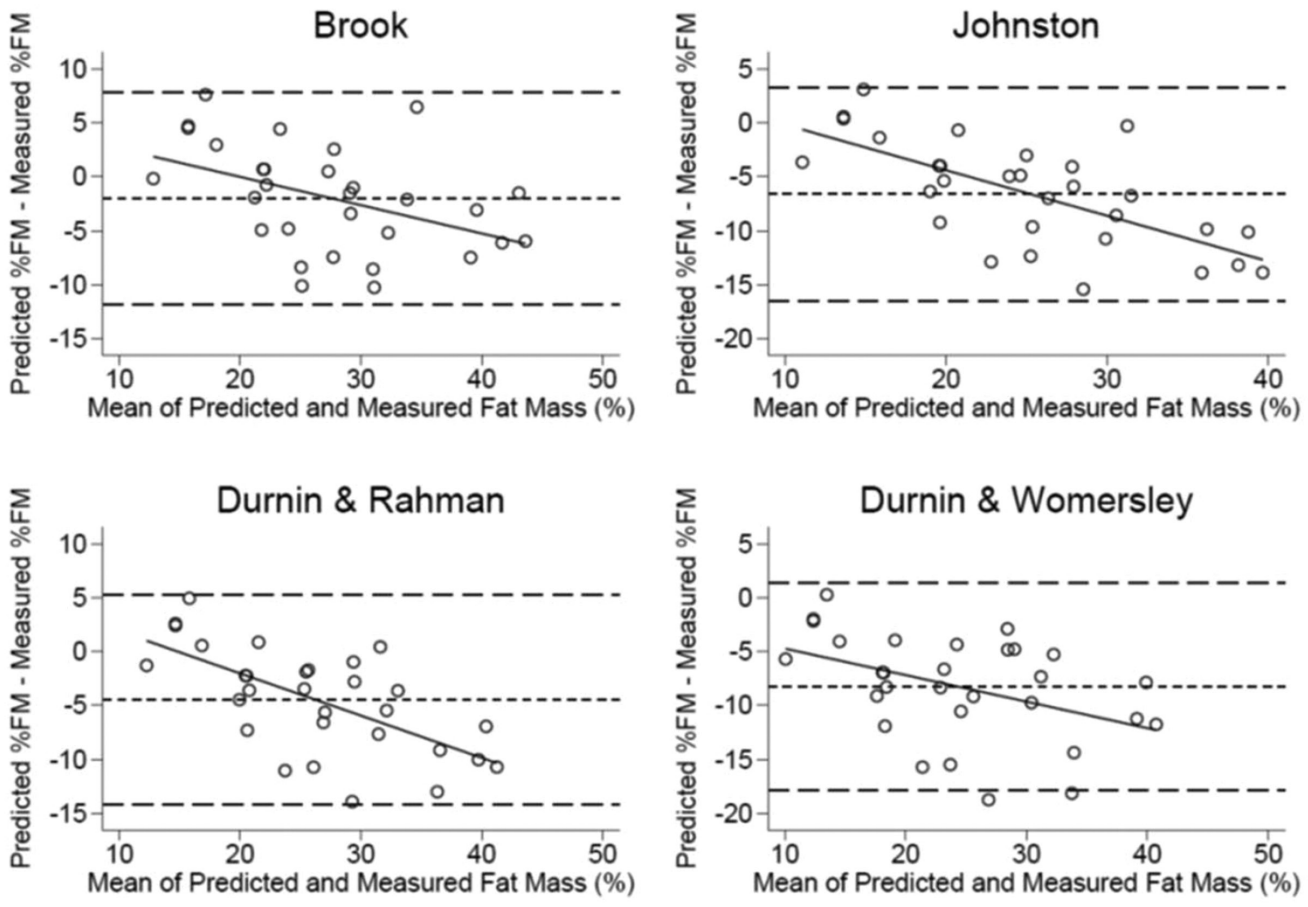
Bland and Altman plots for each fat mass percentage predictive equation compared with measured fat mass percentage (dual-energy X-Ray absorptiometry).

When we compared predicted FM% using previous predictive equation, we found that in IBD patients, the Durnin and Womersley equation had the highest median (25th, 75th percentile) percentage bias (−26.4% [−36.7; −16.4%]), followed by Johnston equation (−23.3% [−29.8; −13.6]), Durnin and Rahaman equation (-14.2% [22.3; −6.4%]), and Brook equation (−5.6% [−17.5; 3.3]). Figure 2 shows Bland and Altman plots for each FM% predictive equation compared with measured FM%, revealing a proportional bias affecting all equations (Pitman test *P* < 0.05).

## DISCUSSION

Considering the values of FM% obtained with DEXA technique, no significant differences in terms of FM% was observed between subjects with IBD and HCs (FM% 29.6% vs 32.2%, *P* = 0.108). Five studies evaluated fat free mass (FFM) in patients with CD (n = 255) (25–29). Deficits of FFM were described in 3 studies (n = 221). Sentongo et al (25) described deficits in FFM between CD patients (n = 132) and HCs, using DEXA technique and skinfold measurements after adjustment for age in both male and female individuals. Varille et al (26) also showed deficits in FFM in cohort patients with CD (n = 11) with stricturing refractory disease phenotype, before surgery. Thayu et al (27) assessed whole body composition (FFM and FM) using DEXA technique in 78 CD subjects and 669 HCs, ages 5 to 21 years. FFM was significantly lower in girls with CD compared with controls (*P* < 0.01). Within the boys , FFM was significantly lower in the nonblack subjects with CD compared with controls (*P* < 0.001).

Zoli et al (28) showed no difference in FFM between CD and controls in a small study (n = 10), the patients in this cohort were in remission and skinfold thicknesses were used for estimation of body composition. Azcue et al (29) characterized body composition and resting energy expenditure (REE) in 24 children with CD and compared data with HCs and with female subjects with anorexia nervosa. Body weight, ideal body weight, and FFM were lower in patients with CD than in HCs (29). A possible explanation for reduced FFM is not only hypercatabolism caused by acute inflammation mediated by circulating pro-inflammatory cytokines but also by medications, such as glucocorticoids (30,31).

There are few studies reporting on FM in children with IBD (n = 611, CD 502, UC 109) (25,32–38). The majority of studies used DEXA technique for measurement of FM (n = 516) (28–33), only 2 studies used bioelectrical impedance (n = 95) (37,38). No significant difference was found in terms of FM between IBD patients and HCs except for 2 studies conducted by Thayu et al (27) and Boot et al (34), respectively. In the first study, authors found gender-related differences in body composition deficits at diagnosis in patients with CD. In girls, CD was associated with significantly lower FM (*P* = 0.001), adjusted for age, race, and Tanner stage, compared with HCs. All patients included in the study had moderate-to-severe disease activity. Boot et al (34) evaluated bone mineral density and body composition with DEXA technique of 55 patients with IBD (22 CD and 33 UC). Decreased FM was found in patients with longer disease duration (mean 2.2 years).

In Model 1, we evaluated the contribution of continuous predictors expressed as loge, discrete variables (IBD yes, IBD no) and their interaction in the prediction of FM. The continuous variables that were considered were: BMI (logeBMI), TSF (logeTSF), sum of 2 skinfolds (triceps + biceps loge2SF) and sum of 4 skinfolds (triceps + biceps + subscapular + suprailiac loge4SF).

Figure 1 shows 4 scatter plots that display data of FM in function of the predictors and the interaction between the continuous and discrete variables.

We used 4 prediction models based on anthropometry, for estimation of FM%. Results are shown in Table 2. As determined by *R*^2^_adj_ (adjusted coefficient of determination) and RMSE (root mean square error), Model 1 was the less accurate (*R*^2^_adj_ 0.68, RMSE 4.94%) confirming that BMI is a poor predictor of body composition. Model 3, obtained by incorporating the sum of 2 skinfolds (biceps + triceps) demonstrated a slight improvement in predicting FM % compared with applying only the single triceps fold in Model 2 (*R*^2^_adj_ 0.86, RMSE 3.23% vs *R*^2^_adj_ 0.82, RMSE 3.75%). Model 4, obtained by incorporating the sum of 4 skinfolds (biceps + triceps + suprailiac + subscapular) revealed an additional improvement in prediction of FM% compared with Model 3 (*R*^2^_adj_ 0.88, RMSE 3.03% vs *R*^2^_adj_ 0.86, RMSE 3.23%). We reckon that the proposed method based on skinfold thickness measurements for estimation of FM% is feasible in clinical practice and acceptable for patients as it is a noninvasive procedure. The only critical aspect is that skinfold thicknesses should be measured by trained dietitians that are not always present in all clinical settings.

Our study has some limitations, in first instance the low numerosity of subjects included did not permit to evaluate differences in body composition between patients with CD and UC. In literature, the available studies show no difference in deficits of FFM between CD and UC; however, FM is lower in CD than UC. There is a discrepancy between age and pubertal stage between the 2 groups (IBD and HCs) because of the fact that the groups were comparable for range of age but not matched for age and gender. This could have influenced results on FM%. For this reason, we have performed adjustment for age and sex in the statistical analysis.

Furthermore, an association between body composition and disease activity and treatment has not been performed. There are studies reporting on patients with active disease (n = 160, CD 153, UC 7) (25,33,36,37) with LM deficits. In 2 of these studies, however, patients were receiving systemic steroids at the time of study, and thus this may contribute to these findings. Indeed, there is increasing evidence on the effects of anti-TNFα treatment on body weight and body composition (39–41). This aspect is extremely important as increased body weight is also a risk factor for loss of response to anti-TNF therapy (infliximab and adalimumab). Future studies should attempt to differentiate between the effects of therapy and the disease process itself.

## CONCLUSIONS

Despite the results of our study have not found significant differences in FM% using DEXA technique between subjects with IBD and HCs, it is known that patients with IBD are at increased risk of having altered body composition because of several risk factors including not only malnutrition secondly to the underlying gastrointestinal disease but also pharmacological treatment (corticosteroids, anti-TNFα therapy). Given the importance of nutritional status in these patients, whenever DEXA scan is not available, it is possible to use skinfold thicknesses to estimate FM%. In fact, we have shown that the sum of 4 skinfolds (triceps + biceps + subscapular + suprailiac) is the most accurate in predicting FM% in children and adolescents with IBD. The sum of 2 skinfolds (triceps + biceps) is similarly accurate, in addition, the measurement of 2 skinfolds versus 4 skinfolds could be less prone to measurement error. The newly developed population-specific formulae with the sum of 2 or 4 skinfolds could be a valid tool for estimation of body composition in children with IBD and valid alternative to DEXA measurement. Further prospective studies are needed in order to confirm our data and validate the specific formulae.

## Supplementary Material
