# Supplementary material for: Predictive Fat Mass Equations for Children With Inflammatory Bowel Disease
Source: J Pediatr Gastroenterol Nutr. 2021 Jun 4;73(4):e98–e104. doi: 10.1097/MPG.0000000000003188 (PMC10237347; doi:10.1097/MPG.0000000000003188)
Supplement: Supplementary file 1 [file jpga-73-e98-s001.docx]

| **Diagnosis**  CD 16 (53.3%)  UC 12 (40%)  IBD-U 2 (6.6%) |
| --- |
| **Crohn’s Disease (n=16)**  **Phenotype at diagnosis (Paris classification)**  **Disease location**  ileocolonic (L3) 10/16 (62.5%)  ileal (L1) 3/16 (18.7%)  ileal and upper disease prox to Treitz L1 + L4a 3/1 (18.7%)  **Disease behaviour**  Non stricturing and non- penetrating (B1) 28/30 (93.3%)  Stricturing (B2) 2/30 (6.6%)  **Growth delay**  No growth delay (G0) 29/30 (96.6%)  Growth delay (G1) 1/30 (3.3%)  **Disease activity at enrollment**  Remission (sPCDAI <15) 10/16 (62.5%)  Mild disease (sPCDAI 15-30) 6/16 (37.5%)  Moderate and Severe activity (sPCDAI 30-90): 0  **Treatment**  Azathioprine 11/16 (68.7%)  Infliximab 4/16 (25%)  Corticosteroids 2/16 (12.5%)  Exclusive enteral nutrition 1/16 (6.3%)  Partial enteral nutrition 4/16 (25%) |
| **Ulcerative colitis (n=12)**  **Disease location at diagnosis (Paris Classification)**  Pancolitis (E4) 8/12 (66.6%)  Left-sided UC (E2) 2/12 (16.6%)  Proctosigmoiditis (E1) 2/12 (16.6%)  **Disease activity at enrollment**  Remission (PUCAI<10) 9/12 (75%)  Mild activity (PUCAI 10-35) 3/12 (25%)  Moderate activity (PUCAI 40-60) 0  Severe activity (PUCAI 65-85) 0  **Treatment**  5-aminosalicylates 8/12 (66.6%)  Azathioprine 3/12 (25%)  Colectomy 1/12 (8.4%) |
| **IBD-U (n=2)**  **Treatment:**  Azathioprine 2/2 (100%) |

**Supplementary table.** Data on disease location and activity and treatment in IBD patients.
